# Exploring tight junction alteration using double fluorescent probe combination of lanthanide complex with gold nanoclusters

**DOI:** 10.1038/srep32218

**Published:** 2016-08-30

**Authors:** Xinyi Wang, Na Wang, Lan Yuan, Na Li, Junxia Wang, Xiaoda Yang

**Affiliations:** 1 Department of Chemical Biology, School of Pharmaceutical Sciences, Peking University, Beijing 100191, China; 2 College of Sciences, Shenyang Agricultural University, Shenyang 110161, China; 3 Department of Pharmacognosy, School of Pharmaceutical Sciences, Hebei Medical University, Shijiazhuang 050017, China; 4 Beijing National Laboratory for Molecular Sciences (BNLMS), The Key Laboratory of Bioorganic Chemistry and Molecular Engineering, Ministry of Education, College of Chemistry and Molecular Engineering, Peking University, Beijing 100871, China; 5 Department of Molecular Biology, Hebei Key Lab of Laboratory Animal, Hebei Medical University, Shijiazhuang 050017, China

## Abstract

Tight junctions play a key role in restricting or regulating passage of liquids, ions and large solutes through various biological barriers by the paracellular route. Changes in paracellular permeation indicate alteration of the tight junction. However, it is very difficult to obtain the structural change information by measuring paracellular flux based on transepithelial electrical resistance or using fluorescein-labeled dextrans. Here we show that the BSA and GSH stabilized gold nanoclusters exhibit marginal cytotoxicity and pass through the MDCK monolayer exclusively through the paracellular pathway. We propose a double fluorescence probe strategy, the combination of a proven paracellular indicator (europium complex) with fluorescent gold nanoclusters. We calculate changes of structural parameters in tight junctions based on determination of the diffusion coefficients of the probes. Two different types of tight junction openers are used to validate our strategy. Results show that EDTA disrupts tight junction structures and induces large and smooth paracellular pore paths with an average radius of 17 nm, but vanadyl complexes induce paths with the radius of 6 nm. The work suggests that the double fluorescence probe strategy is a useful and convenient approach for *in vitro* investigation of tight junction structural alternations caused by pharmacological or pathological events.

The tight junction (TJ), located at the apical-most portion of the intercellular junctional complex, composes over a hundred TJ proteins arranged in order and fabricates a complex signaling network involving trans-membrane proteins, cytoskeletal proteins, scaffolding components, regulatory and signaling molecules, etc^1^,^2^. TJ plays a key role in restricting or regulating passage of liquids, ions, and large solutes through the paracellular pathway^3^,^4^,^5^. TJ structures can be altered under various physiological and/or pathological events, to name a few, the salivary gland and some endothelial cell secretion^6^,^7^, inflammatory mediator-induced changes in paracellular permeability^8^,^9^ and toxicity of metal ions^10^,^11^. Moreover, the use of TJ opening reagents^12^,^13^,^14^,^15^,^16^,^17^, e.g. chitosan^18^,^19^,^20^,^21^, has been an important biopharmaceutical strategy for the delivery of highly hydrophilic macromolecular drugs (e.g. peptide/protein drugs). Thence, methods that help to characterize the properties of paracellular pathways would be of significance for elucidating the TJ architecture and mechanism of regulation.

The TJ structure can be visualized by electron microscopy^22^,^23^ or fluorescence microscopy with genetically encoded fluorescent proteins^24^,^25^, however, these approaches are not feasible to study the alteration of the tight junction upon the treatment with drugs and/or stimuli. This TJ alteration is readily indicated by the changes of the trans-epithelial electrical resistance (TEER)^26^,^27^ or the apparent permeability coefficient (*P*_app_) of a paracellular permeation probe/indicator^28^,^29^, e.g. manitol^30^,^31^, inulin^32^,^33^, cimetidine^34^,^35^ and hydrophilic fluorophores such as Eu-DTPA complexes^36^,^37^, lucifer yellow^38^,^39^ and different sized fluorescein isothiocyanate-labeled dextrans (MW 3∼40 kDa, e.g. *FD4*)^40^,^41^. However, TEER cannot provide any information about the TJ pore size or charge selectivity; while the previous approaches using paracellular probes/markers would either provided very limited information and/or require abundant of work. It is a great challenge to develop new methods that can be easily and conveniently implemented to sense the TJ changes associated with the physiological and/or pathological events.

Previous studies using the freeze-fracture technique and probes of different sizes revealed that the TJ structure had several pore pathways allowing flux of solutes with radii ranging from about 4 Å to 60 Å^42^. This inspires us to postulate that the diffusion of solutes in the paracellular routes could be similar to that in the porous medium. Therefore, it is possible to calculate the pore size (*r*) and retention parameters of tight junction channels by measuring the diffusion coefficients (*D*_p_) of two or more probes of known molecular sizes and following the Fick’s law of diffusion and the amended Knudsen equation.

In the present work, we developed a novel method for investigating the dynamic change of the TJ pore size (*r*) and the channel retention property. Two sized fluorescent gold nanoclusters (AuNCs), BSA-templated AuNCs (AuNC@BSA) and GSH-protected AuNCs (AuNC@GSH), were first validated as novel paracellular permeability indicators. Then by combining the proven paracellular indicator, Eu-DTPA complex, with one of the AuNCs, the change of the TJ pore size (*r*) and retention parameters upon treatment of two different TJ openers was calculated.

## Results and Discussion

### Synthesis and characterization of AuNCs

**Figure 1:**
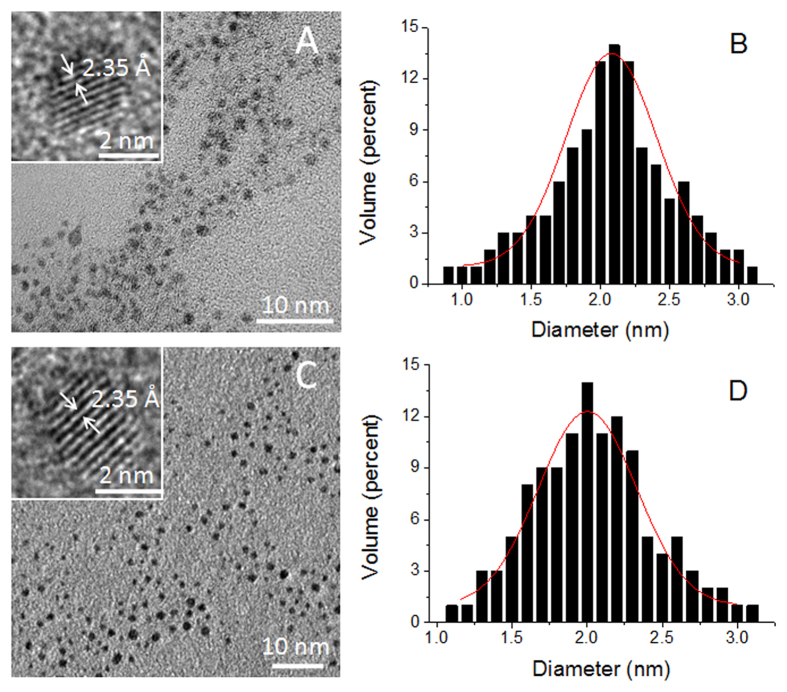
TEM images of AuNC@GSH (**A**) and AuNC@BSA (**C**). Insets display thelattice fringe of an individual nanocluster. Histograms of (**B**) and (**D**) show the size distribution of AuNC@GSH and AuNC@BSA obtained from corresponding TEM image, respectively.

AuNC@GSH^43^ and AuNC@BSA^44^ were prepared according to the published procedure. Characterization using UV-Vis, fluorescence, infrared, X-ray photoelectron spectroscopies and DLS (Fig. S1–S5) demonstrated that the synthesis resulted in desired products. TEM measurements indicated that the core size for AuNC@GSH and AuNC@BSA were 2.07 ± 0.45 nm (*n* = 120) and 2.04 ± 0.40 nm (*n* = 120), respectively (Fig. 1). Insets in TEM images show lattice plane parameters match the (111) lattice spacing of the face-centered cubic Au^45^. In DMEM cell culture media, AuNC@GSH and AuNC@BSA (Fig. S5) showed the volume weighted hydrodynamic sizes of 6.0 ± 1.0 nm and 12.1 ± 0.3 nm, respectively. Both AuNCs had negative surface charges (Table 1).

### Cytotoxicity of AuNCs on MDCK cells

**Figure 2:**
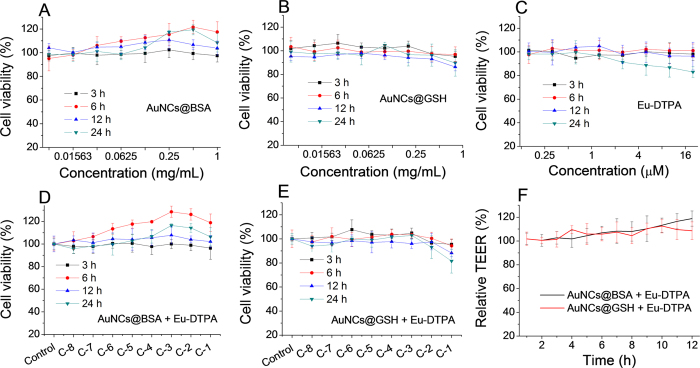
MTS assays of MDCK cells upon treatment with AuNCs and Eu-DTPA. (**A**) AuNC@BSA. (**B**) AuNC@GSH. (**C**) Eu-DTPA. (**D**) AuNC@BSA + Eu-DTPA. (**E**) AuNC@GSH + Eu-DTPA. C-1 denotes combination of 20 μmol/L Eu-DTPA with 1 mg/mL AuNC@BSA or 0.8 mg/mL AuNC@GSH, and C-2∼C-8 denote proportional dilution of C-1. (**F**) TEER of MDCK cell monolayer after treated with AuNCs and Eu-DTPA combination at C-1 concentration. All data were the mean ± SD of four replicates.

The cytotoxicity of AuNCs and Eu-DTPA complexes on MDCK cell was assessed by MTS assays and TEER measurements. Results (Fig. 2) showed that AuNCs or Eu-DTPA complexes or their combination had no significant effects on cell viability in the test concentration ranges. The cell viability greater than 100% upon treatment with high concentrations of AuNC@BSA (Fig. 2A,D) was an artifact caused by the excessive amount of BSA in AuNC@BSA preparation. The alternation of TEER is normally more sensitive than MTT/MTS assessments^10^, however, it was observed that AuNCs or Eu-DTPA did not cause any decline of TEER over 24-h incubation (Fig. 2F). These results indicated that AuNCs or the combination with Eu-DTPA was not toxic to MDCK cells.

### Mechanism of AuNCs permeation through MDCK monolayer

For calculation of TJ parameter changes, two different size paracellular pathway probes are needed. The Eu-DTPA complex is selected as the small size probe due to good biocompatibility and correlation of *P*_app_ with TEER^46^,^47^. In addition, the long life fluorescence of Eu^3+^ allows the use of time-resolved fluorescence measurements to achieve a high sensitivity with the interference-free background without separation from the second fluorescent probe. The gold nanoclusters (AuNCs) are considered as the large size probes due to their tunable particle size, excellent biocompatibility and desirable photostability^44^,^48^,^49^. In addition, if successful, it may open new opportunities for biological application of fluorescent nanomaterials.

**Figure 3:**
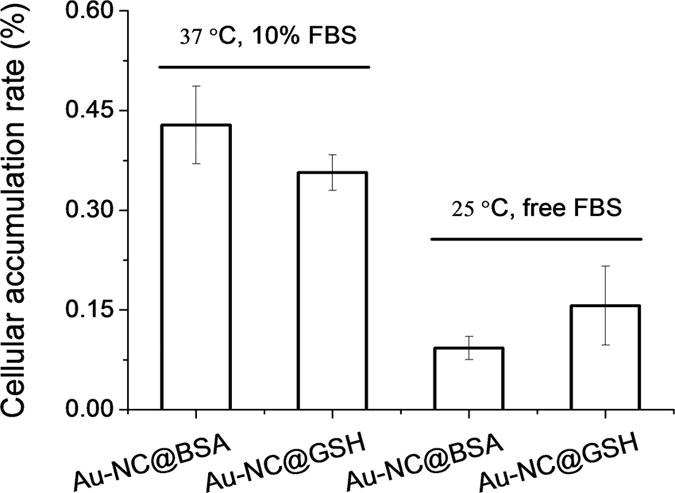
Rates of cellular accumulation of AuNCs in MDCK cells. The incubated time was 6 h at 37 °C (10% FBS) and 25 °C (free FBS), respectively.

**Figure 4:**
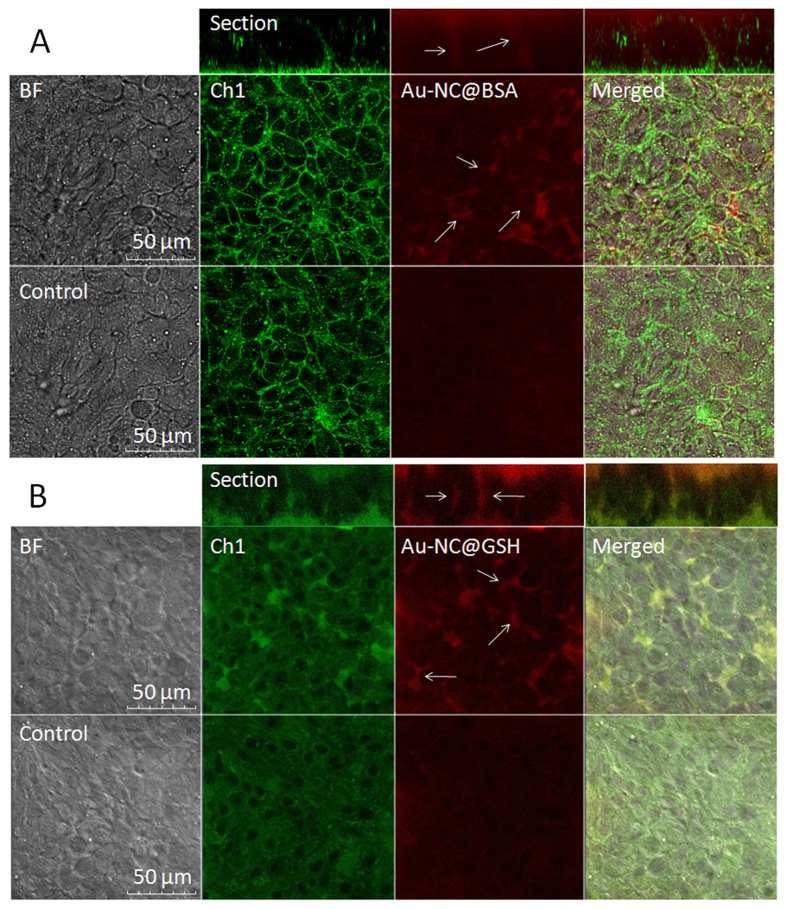
Confocal images of AuNCs in TJs of MDCK layers after EDTA treatment (0.5 mmol/L) for 30 min at 25 °C. The concentrations of AuNCs were 1 mg/mL for AuNC@BSA (**A**) and 0.8 mg/mL for AuNC@GSH (**B**). Ch1 denote cell autofluorescence with *λ*_Ex/Em_ of 488/520 nm.

The criteria determining the mechanism of membrane permeation of a solute include the value of the apparent permeability coefficient (*P*_app_)(less than 10^−6^ cm/s for paracellular diffusion)^50^ and the rate of cellular accumulation. The *P*_app_ of AuNC@BSA and AuNC@GSH were determined to be 1.99 × 10^−7^ cm/s and 3.66 × 10^−7^ cm/s, respectively. ICP-MS assays indicated (Fig. 3) that cellular accumulation of both AuNCs (<0.5%) were marginal. Moreover, under the confocal fluorescence microscope, AuNCs were only observed in the intercellular space (Fig. 4) upon opening TJ with EDTA. All the evidence indicated the transport of AuNC@BSA across MDCK monolayer exclusively through the paracellular pathway.

### Changes of *P*_app_ of the Eu-DTPA-AuNCs double probes upon TJ opening

The paracellular permeability changes upon TJ opening in response to a variety of physiological and/or toxicological factors, e.g. cytokines, stress factors, dextrans, metal ions and metal chelators. Herein, we tested the effect of two different TJ openers, EDTA and vandylacetylacetonate (VO(acac)_2_). EDTA causes reversible TJ opening by depleting Ca^2+^ and Mg^2+^ that maintains the structure and conformation of TJ proteins^51^,^52^; while, vanadium complexes can increase paracellular permeability through induction of oxidative stress, which was regarded as one of the major mechanism for the toxicity of these anti-diabetic agents^10^.

**Figure 5:**
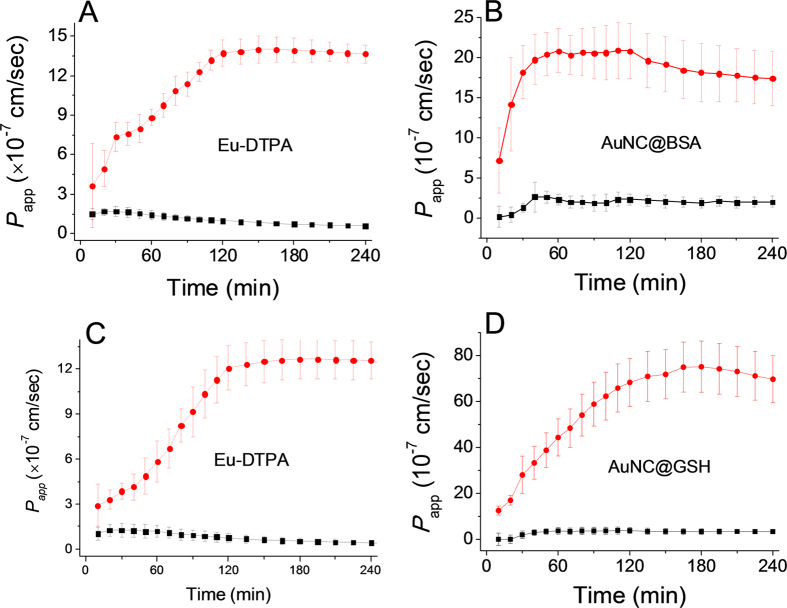
The time course of membrane permeation of double fluorescent probes in absence (black line) and presence (red line) of EDTA treatment (0.5 mmol/L). A and B are the permeability of Eu-DTPA (**A**) and AuNC@BSA (**B**) in the Eu-DTPA (20 μmol/L)−AuNC@BSA (1 mg/mL) probe system, respectively; C and D are the permeability of Eu-DTPA (**C**) and AuNC@GSH (**D**) in the Eu-DTPA (20 μmol/L)−AuNC@GSH (0.8 mg/mL) probe system, respectively. Data were the mean ± SD of three replicates.

**Figure 6:**
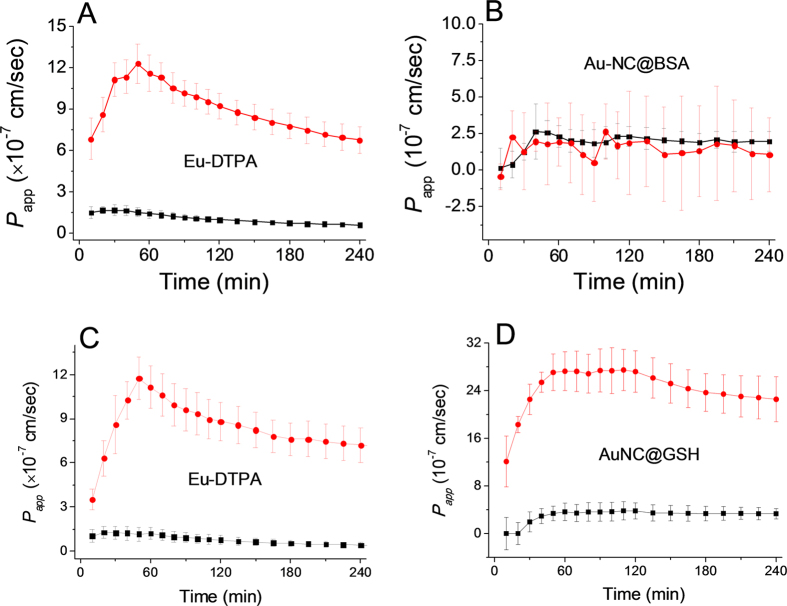
The time course of membrane permeation of double fluorescent probes in absence (black line) and presence (red line) of vanadyl complexes (80 μmol/L). A and B are the permeability of Eu-DTPA (**A**) and AuNC@BSA (**B**) in the Eu-DTPA (20 μmol/L)−AuNC@BSA (1 mg/mL) probe system, respectively; C and D are the permeability of Eu-DTPA (**C**) and AulNC@GSH (**D**) in the Eu-DTPA (20 μmol/L)−AuNC@GSH (0.8 mg/mL) probe system, respectively. Data were the mean ± SD of three replicates.

As shown in Fig. 5, upon addition of EDTA (0.5 mmol/L), the paracellular permeability of the probes increased significantly. Upon treatment with vanadyl complexes(80 μmol/L, Fig. 6), the permeability of Eu-DTPA and AuNC@GSH increased rapidly in the first hour and then gradually decreased, suggesting partial recovery of the TJ structure in the later hours. Treatment with vanadyl complexes did not elevate the permeability of AuNC@BSA. These results indicated that EDTA and vanadyl complexes caused different patterns of TJ alternation in both the extent of opening and the TJ architecture.

### Alternations of TJ pore size and retention capacity upon TJ opening

**Figure 7:**
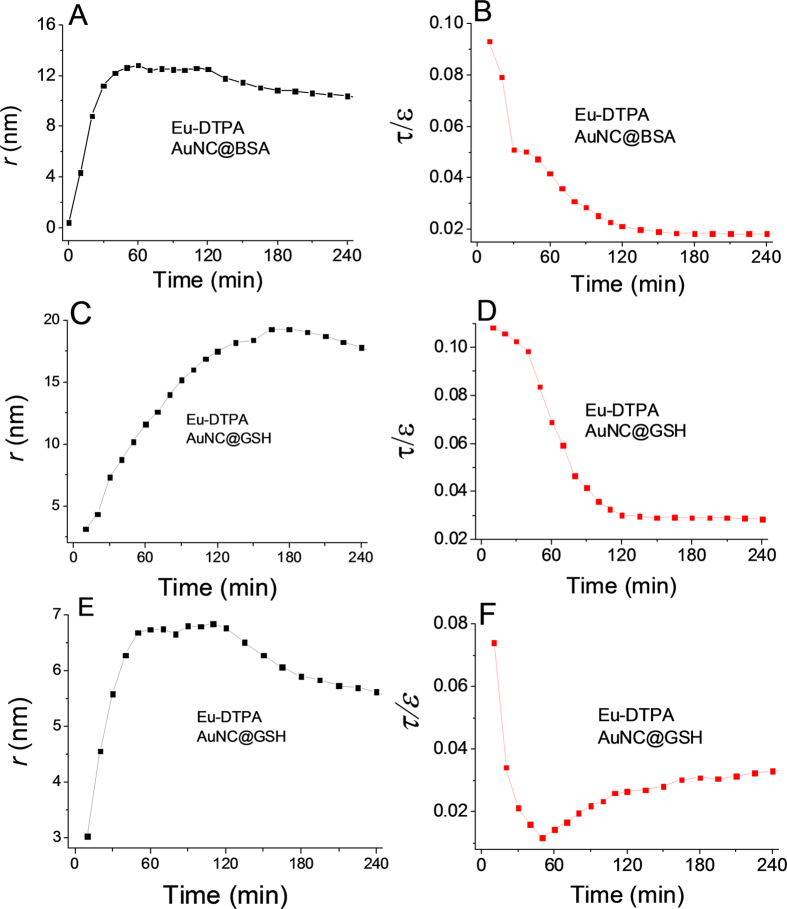
Alteration of tight junction pore size, *r* (left column) and retention capacity, *ε*/*τ* (right column) calculated by use of Eu-DTPA/AuNC@BSA probes (**A,B**) and Eu-DTPA/AuNC@GSH (**C,D**) probes upon EDTA treatment, and Eu-DTPA/AuNC@GSH (**E,F**) probe upon vanadium treatment.

By using the *P*_app_ data in Figs 5 and 6, the pore size (*r*) and retention capacity values (*ε*/*τ*) of the tight junction of MDCK cell monolayer upon treatment with EDTA or VO(acac)_2_ were estimated accordingly (Fig. 7).Several important conclusions can be drawn from the results. First, the EDTA treatment (Fig. 7) caused two major changes: the TJ pore size (*r*) significantly increased to ∼16 nm while the TJ retention capacity (*ε*/*τ*) decreased monotonously. This suggests that depletion of Ca^2+^ by EDTA induced large and smooth pores in the intercellular space possibly by directly disrupting the TJ protein connection. The calculation using two sets of probes fundamentally gave a similar result. Second, as the permeability of AuNC@BSA did not change for the vanadyl complex treatment, the calculation was achieved on the Eu-DTPA -AuNC@GSH probe set. Results (Fig. 7E,F) indicated that the vanadium treatment increased TJ pore size (*r*) up to ∼6 nm while the retention capacity (*ε*/*τ*) was observed to rapidly decrease at first but then recovered partially in the following hour of incubation, which agrees with the change of the permeability of Eu-DTPA probe (Fig. 6A,C). Overall, the results suggested that vanadium may alter the TJ structure possibly by re-arranging the TJ architecture, leading to domination of the large pore path (∼60 Å)^42^ in the intercellular space. Nevertheless, the details of the mechanism need to be further investigated by biological and structural methods. Third, it is noted that the *ε*/*τ* response of AuNC@BSA upon EDTA treatment showed an interrupt point at ∼40 min and the increase of pore size was suspended afterward at ∼12 nm (Fig. 7A,B). While AuNC@BSA assay (Fig. 7C,D) showed a continual increase of pore size up to 17 nm until 180 min of treatment. We postulated that AuNC@BSA, due to the interaction of the template albumin with the pathway molecules, might stay somewhere in the paracellular path and thus restrict further increase of TJ pore. Probably for the same reason, the *P*_app_ of AuNC@BSA did not response to the vanadium treatment (Fig. 6B). The present results were consistent with our previous observation that a BSA-Eu-DTPA complex did not show *P*_app_ correlation to the treatment of vanadyl complexes^53^.Therefore, AuNC@GSH may better reflect alteration of the TJ structure than AuNC@BSA. In addition, these results may suggest that albumin might play a role in repairing TJ damage and as well address the role of protein corona on regulating the biological properties of nanoparticles^54^,^55^. Additionally, although TEER assays indicate <20% change in TJ upon the treatment of Caco-2 or MDCK cells with vanadium compounds herein and in our previous studies, the pore size caused by vanadium compounds was found up to 6 nm, which should allow flux of most biological substances. Therefore, more attention should be paid to the metal toxicity on TJ of biological barriers in further toxicological studies of vanadium compounds.

## Conclusions

In summary, we have demonstrated that a double fluorescent probe strategy can be used to study the alteration of TJ structure. Our work first confirms that AuNCs pass through the MDCK monolayer exclusively through the paracellular pathway. Then, we successfully investigated the kinetic properties of TJ structure changes for the first time by employing the double fluorescence probes of Eu-DTPA and AuNCs following treatment with two different TJ openers. The calculation suggests that EDTA treatment induces large and smooth pore path with the pore size of ∼17 nm, while vanadyl complexes cause TJ structure re-arrangement and induce TJ pores with pore size of ∼6 nm. Overall, our work indicates that combination of Eu-DTPA and AuNCs probes may provide new and convenient tools to investigate TJ structural alternations *in vitro* in pharmacological and/or pathological processes.

## Methods

### Synthesis of gold nanoclusters (AuNCs)

GSH-protected gold nanoclusters (denoted as AuNC@GSH) were synthesized according to the previous method^43^. Briefly, 10 mL of HAuCl_4_ solution (4 mmol/L) was mixed with 10 mL of glutathione solution (6 mmol/L) under vigorously stirring. Then the mixture was heated up to 70 °C and incubated for 24 h under continuous gentle stirring. The excessive amounts of glutathione and unreacted-HAuCl_4_ were removed by dialysis and the concentration of product AuNC@GSH was quantified using ICP-MS(NexlON 300X, Perkin Elmer, USA).

BSA-stabilized AuNCs (denoted as AuNC@BSA) were prepared according to the previously reported procedure^44^. Briefly, a solution of HAuCl_4_ (10 mmol/L) was added to an equal volume of BSA solution (50 mg/mL). The mixture was vigorously stirred for 2 min, followed by adding 1/10 volume of 1 M NaOH. The resulting mixture was incubated at 37 °C under nitrogen purging and continuous gentle stirring (ca. 200 rpm) for 12 h. The concentration of AuNC@BSA solution was quantified using ICP-MS (NexlON 300X, Perkin Elmer, USA) after purification by dialysis and stored at 4 °C.

### Preparation of Eu-DTPA probe

Eu-DTPA complexes were prepared using the previous method^56^. Briefly, 0.01 mol/L EuCl_3_ solution (prepared by dissolving 0.2760 g Eu_2_O_3_ in 5 mL of 3 mol/L HCl and diluting to 100 mL with double-distilled H_2_O) was added dropwise into 0.01 mol/L DTPA solution in Hank’s balanced salt solution (HBSS; pH 7.0) until the appearance of a white cloudy sediment, then the supernatant (Eu-DTPA) was collected after centrifuge (3 min, 10000×g).

### Preparation of non-fluorescence DMEM medium (NF-DMEM)

To prevent the interference of auto-fluorescence background in DMEM media, some amino acids (tryptophan, Tyrosine, and Phenylalanine) and vitamins (folic acid, pyridoxine hydrochloride, and riboflavin etc.) were excluded referring to GIBCO^TM^ catalogue media formulations when preparing the non-fluorescence DMEM media (NF-DMEM). The fluorescence-free DMEM media did not show any influences on MDCK cell viability or tight junction formation of MDCK cell monolayer.

### MDCK cell culture

MDCK cells were cultured in high glucose DMEM supplied with 10% FBS and penicillin-streptomycin (100 μg/mL) and maintained in a humidified atmosphere containing 5% CO_2_ at 37 °C in 25 cm^2^ plastic flasks. The medium was refreshed every 2 days. Cells were passaged at 70–90% confluency using 0.25% (w/v) trypsin-0.02% (w/v) ethylenediaminetetraacetic acid (EDTA) solution. The MDCK cells used in this study were under passage 50.

### Cytotoxicity assay

Toxicity of AuNCs on MDCK cells was estimated by MTS assay using a Cell Titer 96^®^ A Queous One Solution Cell Proliferation Assay kit (Promega Inc., Madison, WI). In brief, MDCK cells (3∼5 × 10^3^ cells/mL) were seeded in 96-well plates (200 μL per well). After attaching on the wall of plates, the cells were incubated with various concentrations of AuNCs in DMEM for 3, 6, 12, and 24 h. Then the cells were rinsed with DMEM and incubated with MTS solution for another 3 h at 37 °C. The absorbance of the color development in AuNCs-treated and -untreated cells was measured in a Bio-Rad Microplate reader.

### Transport experiment and *P*_app_ calculation

Cells were seeded onto Transwell filters (aperture, 3 μm; diameter, 12 mm) at a density of 1 × 10^5^ cells per well and were allowed to grow and differentiate for about 7 days. Cell monolayers were used when the net trans-epithelial electrical resistance (TEER) exceeded 200 Ω∙cm^2^.

For the trans-epithelial transport experiments^10^, the MDCK monolayers in the transwells insert were rinsed twice with pre-warmed NF-DMEM media. Then different concentrations of Eu-DTPA and/or AuNCs in NF-DMEM media were added to the apical chamber and samples were withdrawn from the basolateral chamber at different time intervals. The apparent permeability coefficients (*P*_app_) of Eu-DTPA/AuNCs were calculated by the formula *P*_app_ = (Δ*Q*/Δ*t*)/(*AC*_0_), where *A* is the surface area of the cell monolayers (1.13 cm^2^ in this study), *C*_0_ is the initial concentration of AuNCs (mg/mL) in the apical chamber.

The concentration of Eu-DTPA was determined by a time-resolved fluorescence assay. Briefly, the sample were mixed with two volumes of fluorescence enhancement solution containing 30 μmol/L β-NTA, 10 mmol/L TOPO, 0.2% Triton X-100, and 0.1 mol/L potassium hydrogen phthalate buffer (pH 3.0). After the mixture was sit for 1 h at room temperature, the fluorescence intensity was measured on a *Flexstation 3* microplate reader with *λ*_Ex/Em_ of 340/616 nm and a measurement window from 600 to 1000 ms.

The concentration of AuNCs was determined by fluorescence assays. The fluorescence spectroscopy of AuNCs was obtained with a Hitachi F4600 spectrophotometer (Hitachi, Japan). The fluorescent intensity of AuNC samples from the basolateral chamber were detected on a *Flexstation 3* microplate reader with *λ*_Ex/Em_ of 488/630 nm for AuNC@BSA and 405/608 nm for AuNC@GSH.

### Treatment of MDCK monolayers with EDTA or vanadium complexes

For the paracellular diffusion, permeability of solutes would be greatly improved when the TJ structure was opened or destroyed. EDTA could disrupt TJ proteins by depleting Ca^2+^ and Mg^2+^ ions^57^,^58^ and vanadium complexes can damage TJ through inducing oxidative stress^10^,^59^,^60^. Hereby, the influence of EDTA and vanadium complexes on the permeability of AuNCs and Eu-DTPA across MDCK cell monolayer were investigated by including 0.5 mmol/L of EDTA or 80 μmol/L of VO(acac)_2_ in the Ca^2+^ and Mg^2+^ free NF-DMEM in the transport experiments described above.

### Calculation of the pore size and the retention capacity of tight junction upon TJ opening

Given that the diffusion of the probes in tight junction channels is similar to that in porous medium and there is no specific interaction among the solutes, solvents and tight junction channels, diffusion of the probes could be described by the amended Knudsen equation:


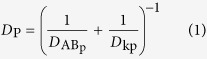


in which


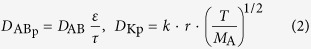


where *D*_AB_ is the diffusion coefficient of probes in solvent B; *ε* and *τ* represent porosity and tortuosity of the porous medium, respectively; *r* is the radius of the pore; *M*_A_ is the molecular mass of the solute. The diffusion coefficient of solutes (*D*_p_) in the paracellular pore pathways can be estimated through the *P*_app_ values (see Supplementary Materials). The *ε*/*τ* is in overall reflecting the retention capacity of the porous medium on the solutes. Considering the size exclusion effect, *ε*/*τ* is thus different among the Eu-DTPA and AuNCs, we assume that the large AuNC probes only pass through the large pores and their *ε*/*τ* is assigned as the intrinsic one. Therefore, the *ε*/*τ* factor for Eu-DTPA would be described by


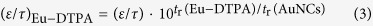


where the retention time (*t*_r_) of the probes was measured on a size exclusion gel column (e.g. Sephadex G25, see Fig. S6). Then, the Knudsen equations for the three fluorescent probes were obtained as:


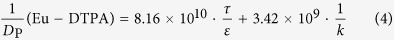



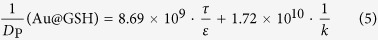



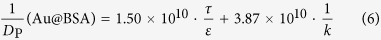


By assigning the size (*r*∼4 Å) of primary TJ pores^42^ as the average size of TJ pores, the calibration constant was calculated to be 0.088 ± 0.008. Then, the change of pore size (*r*) and retention capacity (*ε*/*τ*) could be calculated using the following two sets of equations:

• When using Eu-DTPA + AuNC@GSH:


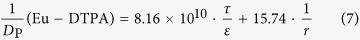



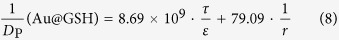


• When using Eu-DTPA + AuNC@BSA:


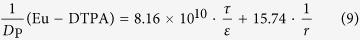



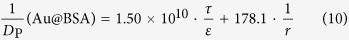


### Confocal Imaging of Transport of AuNCs by the Paracellular Pathway

Briefly, MDCK cells cultured on the glass bottom cell culture dish were rinsed three times and incubated three hours with NF-DMEM media before assay. The detection began after addition of AuNC probes and the TJ opener, EDTA (0.5 mmol/L), in NF-DMEM. The fluorescence was observed with a Zeiss LSM 760 laser scanning confocal microscope (Zeiss, Germany).

### ICP-MS quantifying cellular uptake

When the cell growth reached nearly 90% confluence (in the 6-well microplate), the Eu-DTPA and AuNC probe solution was added into the cultivation media at the desired concentrations at 37 °C (10% FBS) or 25 °C (free FBS). After 6 hours of incubation, the cells were washed, trypsinized and collected. The samples were treated with aqua regia overnight to dissolve the cells and the Au particles. Then the samples were analyzed on an ICP-MS (NexlON 300X, PerkinElmer, USA) to measure the amount of gold atoms per cell.

### Statistics

All the experiments were repeated at least three times. Results are expressed as the means ± SD. All results were analyzed by *t*-test or one-way analysis of variance (ANOVA). A *P*-value less than 0.05 was considered as statistically significant.

## Additional Information

**How to cite this article**: Wang, X. *et al.* Exploring tight junction alteration using double fluorescent probe combination of lanthanide complex with gold nanoclusters. *Sci. Rep.* **6**, 32218; doi: 10.1038/srep32218 (2016).

## Supplementary Material

Supplementary Information

## Figures and Tables

**Table 1:** Some physicochemical parameters of Eu-DTPA and AuNC probes.

Parameters	Eu-DTPA	AuNC@GSH	AuNC@BSA
MA (g/mol)	560.54	1.4 × 10^4^	7.1 × 10^4^
Particle core diameter (nm) (TEM)	—	2.1 ± 0.5	2.0 ± 0.4
Volume weighted hydrodynamic diameter (nm)	0.90^2^	6.0 ± 1.0^3^	12.1 ± 0.3^3^
ζ potential (mV)	—	−1.47 ± 4.96	−7.89 ± 4.38
*D*_AB_ (m^2^/S)^1^	3.36 × 10^−10^	1.15 × 10^−10^	6.68 × 10^−11^

^1^Parameters calculated using the Polson’s equation: 
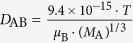
 where*V*_A_ is the volume of solute probe; *μ*_B_ and *M*_B_ are the viscosity and molar mass of the solvent water, respectively; *T* is the absolute temperature.

^2^The diameter of Eu-DTPA was calculated using HyperChem software.

^3^Data were from DLS measurement (Figure S5).

## References

[b1] Tsukita S., Furuse M. & Itoh M. Multifunctional strands in tight junctions. Nat Rev Mol Cell Bio 2, 285–293 (2001). 11283726 10.1038/35067088

[b2] Gonzalez-Mariscal L., Betanzos A., Nava P. & Jaramillo B. E. Tight junction proteins. Prog Biophys Mol Biol 81, 1–44 (2003). 12475568 10.1016/s0079-6107(02)00037-8

[b3] Madara J. L. Regulation of the movement of solutes across tight junctions. Annu Rev Physiol 60, 143–159 (1998). 9558458 10.1146/annurev.physiol.60.1.143

[b4] Wolburg H. & Lippoldt A. Tight junctions of the blood-brain barrier: Development, composition and regulation. Vascul Pharmacol 38, 323–337 (2002). 12529927 10.1016/s1537-1891(02)00200-8

[b5] Shin K., Fogg V. C. & Margolis B. Tight junctions and cell polarity. Annu Rev Cell Dev Biol 22, 207–235 (2006). 16771626 10.1146/annurev.cellbio.22.010305.104219

[b6] Nguyen D. A. D. & Neville M. C. Tight junction regulation in the mammary gland. J Mammary Gland Biol Neoplasia 3, 233–246 (1998). 10819511 10.1023/a:1018707309361

[b7] Ewert P. *et al.* Disruption of tight junction structure in salivary glands from sjogren’s syndrome patients is linked to proinflammatory cytokine exposure. Arthritis Rheum 62, 1280–1289 (2010). 20131287 10.1002/art.27362

[b8] Woywodt A. *et al.* Mucosal cytokine expression, cellular markers and adhesion molecules in inflammatory bowel disease. Eur J Gastroenterol Hepatol 11, 267–276 (1999). 10333199 10.1097/00042737-199903000-00010

[b9] Fasano A. Zonulin and its regulation of intestinal barrier function: the biological door to inflammation, autoimmunity, and cancer. Physiol Rev 91, 151–175 (2011). 21248165 10.1152/physrev.00003.2008

[b10] Zhang Y., Yang X. D., Wang K. & Crans D. C. The permeability and cytotoxicity of insulin-mimetic vanadium (III, IV, V)-dipicolinate complexes. J Inorg Biochem 100, 80–87 (2006). 16321441 10.1016/j.jinorgbio.2005.10.006

[b11] Calabro A. R., Gazarian D. I. & Barile F. A. Effect of metals on beta-actin and total protein synthesis in cultured human intestinal epithelial cells. J Pharmacol Toxicol Methods 63, 47–58 (2011). 20452446 10.1016/j.vascn.2010.04.012 PMC2950867

[b12] Aungst B. J. Intestinal permeation enhancers. J Pharm Sci 89, 429–442 (2000). 10737905 10.1002/(SICI)1520-6017(200004)89:4<429::AID-JPS1>3.0.CO;2-J

[b13] Tamai I. & Tsuji A. Transporter-mediated permeation of drugs across the blood-brain barrier. J Pharm Sci 89, 1371–1388 (2000). 11015683 10.1002/1520-6017(200011)89:11<1371::aid-jps1>3.0.co;2-d

[b14] Salama N. N., Eddington N. D. & Fasano A. Tight junction modulation and its relationship to drug delivery. Adv Drug Del Rev 58, 15–28 (2006). 10.1016/j.addr.2006.01.003 16517003

[b15] Neuwelt E. *et al.* Strategies to advance translational research into brain barriers. Lancet Neurol 7, 84–96 (2008). 18093565 10.1016/S1474-4422(07)70326-5

[b16] Chen Y. & Liu L. Modern methods for delivery of drugs across the blood-brain barrier. Adv Drug Del Rev 64, 640–665 (2012). 10.1016/j.addr.2011.11.010 22154620

[b17] Pardridge W. M. Drug transport across the blood-brain barrier. J Cereb Blood Flow Metab 32, 1959–1972 (2012). 22929442 10.1038/jcbfm.2012.126 PMC3494002

[b18] Sonaje K. *et al.* Opening of epithelial tight junctions and enhancement of paracellular permeation by chitosan: microscopic, ultrastructural, and computed-tomographic observations. Mol Pharm 9, 1271–1279 (2012). 22462641 10.1021/mp200572t

[b19] Hsu L. W. *et al.* Elucidating the signaling mechanism of an epithelial tight-junction opening induced by chitosan. Biomaterials 33, 6254–6263 (2012). 22681978 10.1016/j.biomaterials.2012.05.013

[b20] van der Lubben I. M., Verhoef J. C., Borchard G. & Junginger H. E. Chitosan and its derivatives in mucosal drug and vaccine delivery. Eur J Pharm Sci 14, 201–207 (2001). 11576824 10.1016/s0928-0987(01)00172-5

[b21] Amidi M., Mastrobattista E., Jiskoot W. & Hennink W. E. Chitosan-based delivery systems for protein therapeutics and antigens. Adv Drug Del Rev 62, 59–82 (2010). 10.1016/j.addr.2009.11.009 19925837

[b22] Furuse M. Molecular basis of the core structure of tight junctions. Cold Spring Harb Perspect Biol 2, doi: 10.1101/cshperspect.a002907 (2010). PMC2827901 20182608

[b23] Fujimoto K. Freeze-fracture replica electron microscopy combined with SDS digestion for cytochemical labeling of integral membrane proteins. Application to the immunogold labeling of intercellular junctional complexes. J Cell Sci 108 (Pt 11), 3443–3449 (1995). 8586656 10.1242/jcs.108.11.3443

[b24] Miyamoto T., Furuse M. & Furutani-Seiki M. *In vivo* imaging of tight junctions using claudin-EGFP transgenic medaka. Methods Mol Biol 762, 171–178 (2011). 21717356 10.1007/978-1-61779-185-7_12

[b25] Matsuda M., Kubo A., Furuse M. & Tsukita S. A peculiar internalization of claudins, tight junction-specific adhesion molecules, during the intercellular movement of epithelial cells. J Cell Sci 117, 1247–1257 (2004). 14996944 10.1242/jcs.00972

[b26] Smith J., Wood E. & Dornish M. Effect of chitosan on epithelial cell tight junctions. Pharm Res 21, 43–49 (2004). 14984256 10.1023/b:pham.0000012150.60180.e3

[b27] Tang V. W. & Goodenough D. A. Paracellular ion channel at the tight junction. Biophys J 84, 1660–1673 (2003). 12609869 10.1016/S0006-3495(03)74975-3 PMC1302736

[b28] Suzuki H. *et al.* Crystal structure of a claudin provides insight into the architecture of tight junctions. Science 344, 304–307 (2014). 24744376 10.1126/science.1248571

[b29] Madara J. L. Regulation of the movement of solutes across tight junctions. Annu Rev Physiol 60, 143–159 (1998). 9558458 10.1146/annurev.physiol.60.1.143

[b30] Karlsson P. C., Hughes R., Rafter J. J. & Bruce W. R. Polyethylene glycol reduces inflammation and aberrant crypt foci in carcinogen-initiated rats. Cancer Lett 223, 203–209 (2005). 15896454 10.1016/j.canlet.2004.10.029

[b31] Joh T. *et al.* The protective effect of rebamipide on paracellular permeability of rat gastric epithelial cells. Aliment Pharmacol Ther 18 Suppl 1, 133–138 (2003). 12925151 10.1046/j.1365-2036.18.s1.15.x

[b32] Jaeschke H., Trummer E. & Krell H. Increase in biliary permeability subsequent to intrahepatic cholestasis by estradiol valerate in rats. Gastroenterology 93, 533–538 (1987). 3609663 10.1016/0016-5085(87)90916-4

[b33] Behrens I., Stenberg P., Artursson P. & Kissel T. Transport of lipophilic drug molecules in a new mucus-secreting cell culture model based on HT29-MTX cells.Pharm Res 18, 1138–1145 (2001). 11587485 10.1023/a:1010974909998

[b34] Pade V. & Stavchansky S. Estimation of the relative contribution of the transcellular and paracellular pathway to the transport of passively absorbed drugs in the Caco-2 cell culture model. Pharm Res 14, 1210–1215 (1997). 9327450 10.1023/a:1012111008617

[b35] Nagahara N., Tavelin S. & Artursson P. Contribution of the paracellular route to the pH-dependent epithelial permeability to cationic drugs. J Pharm Sci 93, 2972–2984 (2004). 15459946 10.1002/jps.20206

[b36] Jorgensen V. L., Nielsen S. L., Espersen K. & Perner A. Increased colorectal permeability in patients with severe sepsis and septic shock. Intensive Care Med 32, 1790–1796 (2006). 16964483 10.1007/s00134-006-0356-6

[b37] Hulsmann A. R. *et al.* Permeability of human isolated airways increases after hydrogen peroxide and poly-L-arginine. Am J Respir Crit Care Med 153, 841–846 (1996). 8564141 10.1164/ajrccm.153.2.8564141

[b38] Del Vecchio G. *et al.* Sodium caprate transiently opens claudin-5-containing barriers at tight junctions of epithelial and endothelial cells. Mol Pharm 9, 2523–2533 (2012). 22827574 10.1021/mp3001414

[b39] Akbari P. *et al.* Galacto-oligosaccharides Protect the Intestinal Barrier by Maintaining the Tight Junction Network and Modulating the Inflammatory Responses after a Challenge with the Mycotoxin Deoxynivalenol in Human Caco-2 Cell Monolayers and B6C3F1 Mice. J Nutr 145, 1604–1613 (2015). 26019243 10.3945/jn.114.209486

[b40] Makhlof A., Werle M., Tozuka Y. & Takeuchi H. A mucoadhesive nanoparticulate system for the simultaneous delivery of macromolecules and permeation enhancers to the intestinal mucosa. J Control Release 149, 81–88 (2011). 20138935 10.1016/j.jconrel.2010.02.001

[b41] Benediktsdottir B. E., Gudjonsson T., Baldursson O. & Masson M. N-alkylation of highly quaternized chitosan derivatives affects the paracellular permeation enhancement in bronchial epithelia *in vitro*. Eur J Pharm Biopharm 86, 55–63 (2014). 23608635 10.1016/j.ejpb.2013.04.002

[b42] Shen L., Weber C. R., Raleigh D. R., Yu D. & Tumer J. R. in Annu Rev Physiol 283–309 (Annual Reviews, Palo Alto, 2011). 20936941 10.1146/annurev-physiol-012110-142150 PMC4655434

[b43] Luo Z. T. *et al.* From Aggregation-Induced Emission of Au(I)-Thiolate Complexes to Ultrabright Au(0)@Au(I)-Thiolate Core-Shell Nanoclusters. J Am Chem Soc 134, 16662–16670 (2012). 22998450 10.1021/ja306199p

[b44] Xie J. P., Zheng Y. G. & Ying J. Y. Protein-Directed Synthesis of Highly Fluorescent Gold Nanoclusters. J Am Chem Soc 131, 888–889 (2009). 19123810 10.1021/ja806804u

[b45] Wang C., Hu Y. J., Lieber C. M. & Sun S. H. Ultrathin Au nanowires and their transport properties. J Am Chem Soc 130, 8902–8903 (2008). 18540579 10.1021/ja803408f

[b46] Clausen A. E., Kast C. E. & Bernkop-Schnurch A. The role of glutathione in the permeation enhancing effect of thiolated polymers. Pharm Res 19, 602–608 (2002). 12069161 10.1023/a:1015345827091

[b47] Brayden D. J., Bzik V. A., Lewis A. L. & Illum L. CriticalSorb promotes permeation of flux markers across isolated rat intestinal mucosae and Caco-2 monolayers. Pharm Res 29, 2543–2554 (2012). 22638869 10.1007/s11095-012-0785-6

[b48] Zheng J., Nicovich P. R. & Dickson R. M. In Annu Rev Phys Chem 409–431 (Annual Reviews, Palo Alto, 2007). 17105412 10.1146/annurev.physchem.58.032806.104546 PMC2735021

[b49] Akola J., Walter M., Whetten R. L., Hakkinen H. & Gronbeck H. On the structure of thiolate-protected Au-25. J Am Chem Soc 130, 3756–3757 (2008). 18321117 10.1021/ja800594p

[b50] Gao J. *et al.* Current protocols in pharmacology. John Wiley & Sons, Inc. New York, 7.2.1–7.2.23 (2000).

[b51] Liang X. L. *et al.* Transport properties of puerarin and effect of Radix Angelicae Dahuricae extract on the transport of puerarin in Caco-2 cell model. J Ethnopharmacol 144, 677–682 (2012). 23085309 10.1016/j.jep.2012.10.011

[b52] Lam C. H., Hansen E. A., Janson C., Bryan A. & Hubel A. The characterization of arachnoid cell transport II: paracellular transport and blood-cerebrospinal fluid barrier formation. Neuroscience 222, 228–238 (2012). 22814001 10.1016/j.neuroscience.2012.06.065

[b53] Xu Z. H., Zhang C. Y., Zhang Y. & Yang X. D. Europium Complexes as Novel Indicators of Paracellular Diffusion. Chem biodivers 9, 1916–1922 (2012). 22976980 10.1002/cbdv.201100439

[b54] Monopoli M. P., Aberg C., Salvati A. & Dawson K. A. Biomolecular coronas provide the biological identity of nanosized materials. Nat Nanotechnol 7, 779–786 (2012). 23212421 10.1038/nnano.2012.207

[b55] Walkey C. D. & Chan W. C. Understanding and controlling the interaction of nanomaterials with proteins in a physiological environment. Chem Soc Rev 41, 2780–2799 (2012). 22086677 10.1039/c1cs15233e

[b56] Xu Z. H., Zhang C. Y., Zhang Y. & Yang X. D. Europium Complexes as Novel Indicators of Paracellular Diffusion. Chem Biodivers 9, 1916–1922 (2012). 22976980 10.1002/cbdv.201100439

[b57] Quan Y. S. *et al.* Effectiveness and toxicity screening of various absorption enhancers using Caco-2 cell monolayers. Biol Pharm Bull 21, 615–620 (1998). 9657048 10.1248/bpb.21.615

[b58] Rege B. D., Yu L. X., Husain A. S. & Polli J. E. Effect of common excipients on Caco-2 transport of low-permeability drugs. J Pharm Sci 90, 1776–1786 (2001). 11745735 10.1002/jps.1127

[b59] Xu Z., Zhang C., Zhang Y. & Yang X. Europium Complexes as Novel Indicators of Paracellular Diffusion. Chem Biodivers 9, 1916–1922 (2012). 22976980 10.1002/cbdv.201100439

[b60] Yang X. G., Yang X. D., Yuan L., Wang K. & Crans D. C. The permeability and cytotoxicity of insulin-mimetic vanadium compounds. Pharm Res 21, 1026–1033 (2004). 15212169 10.1023/b:pham.0000029293.89113.d5

