# Supplementary material for: Exploring tight junction alteration using double fluorescent probe combination of lanthanide complex with gold nanoclusters
Source: Sci Rep. 2016 Aug 30;6:32218. doi: 10.1038/srep32218 (PMC5004201; doi:10.1038/srep32218)
Supplement: Supplementary Information [file srep32218-s1.pdf]

Electronic Supplementary Information (ESI†)

Exploring tight junction alteration using double fluorescent probe combination of lanthanide complex with gold nanoclusters†

Xinyi Wang[1,2‡], Na Wang[1,4‡], Lan Yuan[1], Na Li[3], Junxia Wang[5], Xiaoda Yang[1]*

[1]Department of Chemical Biology, School of Pharmaceutical Sciences, Peking University, Beijing 100083, China. Fax: +86-10-62015584; Tel: +86-10-82801539; E-mail: xyang@bjmu.edu.cn

[2]College of Sciences, Shenyang Agricultural University, Shenyang 110161, China.

[3]Beijing National Laboratory for Molecular Sciences (BNLMS), The Key Laboratory of Bioorganic Chemistry and Molecular Engineering, Ministry of Education, College of Chemistry and Molecular Engineering, Peking University, Beijing 100871, China

[4]Department of Pharmacognosy, School of Pharmaceutical Sciences, Hebei Medical University, Shijiazhuang 050017, China

[5]Department of Molecular Biology, Hebei Key Lab of Laboratory Animal, Hebei Medical University, Shijiazhuang 050017, China

* Corresponding author.

† Electronic supplementary information (ESI) available.

‡ These authors contributed equally to this work.

Materials

MDCK (*Madin-Darby* canine kidney) cell was obtained from Institute of Materia Medica, Chinese Academy of Medical Sciences, Beijing. Dulbecco's modified Eagle's medium (DMEM), penicillin-streptomycin and fetal bovine serum (FBS) were from GIBCO, Invitrogen Corp. (Carlsbad, CA, USA). Transwell plates (12 wells, pore diameter of 3 μm, polycarbonate) were from Corning Costar (Cambridge, MA). Eu_2O_3 (99.99%), potassium hydrogen phthalate, and diethylenetriaminepentaacetic acid (DTPA) were from Sinopharm Chemical Reagent Corp (Beijing). Chloroauric acid (HAuCl4) and reduced glutathione (GSH) were purchased from Alfa Chemical (UK). Bovine serum albumin (BSA) was obtained from Sigma-Aldrich (Shanghai, China). Trioctyl phosphine oxide (TOPO) was from Sigma-Aldrich (St. Louis, MO, USA). The MTS tetrazolium compound was from Promega Corp. (Madison, WI, USA). All the other reagents were of analytical grade from commercial source.

Characterization of Probes

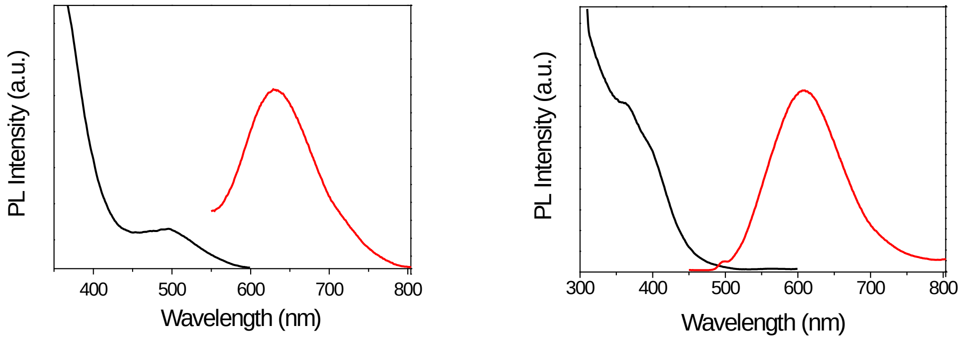

Figure S1. The fluorescence excitation (black lines) and emission (red lines) spectra of (a) AuNC@BSA (λ_{em} = 630 nm for photoexcitation; λ_{ex} = 488 nm for photoemission), (b) AuNC@GSH (λ_{em} = 608 nm for photoexcitation; λ_{ex} = 405 nm for photoemission).

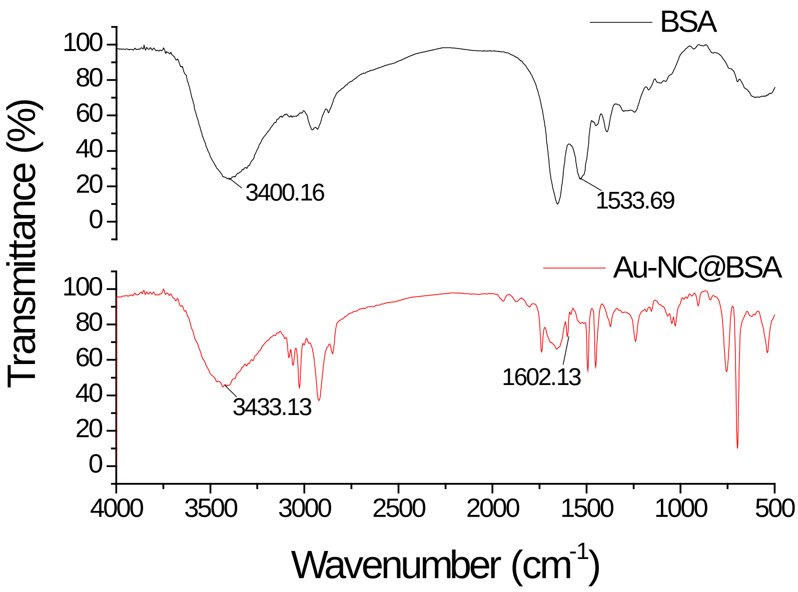

Figure S2. FT-IR spectra of BSA and Au-NCs@BSA. The peaks of 3400.16cm^{-1} and 1533.69 cm^{-1} belong to the C-N stretching vibration and N-H bending vibration of BSA, respectively. The characteristic peak red-shift of BSA in Au-NC@BSA (increased by 33 cm^{-1} and 69 cm^{-1}) indicating the existence of interaction between the amino and Au nanoclusters.

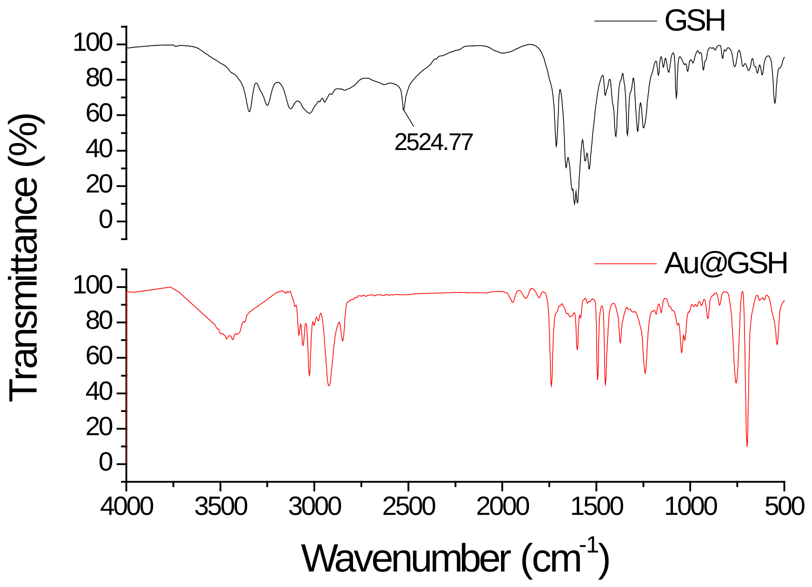

Figure S3. FT-IR spectra of GSH and Au-NCs@GSH. The S-H stretching vibration peak of GSH at 2524.77cm^{-1} disappeared in the product of Au-NC@GSH indicating Au-S bond formation.

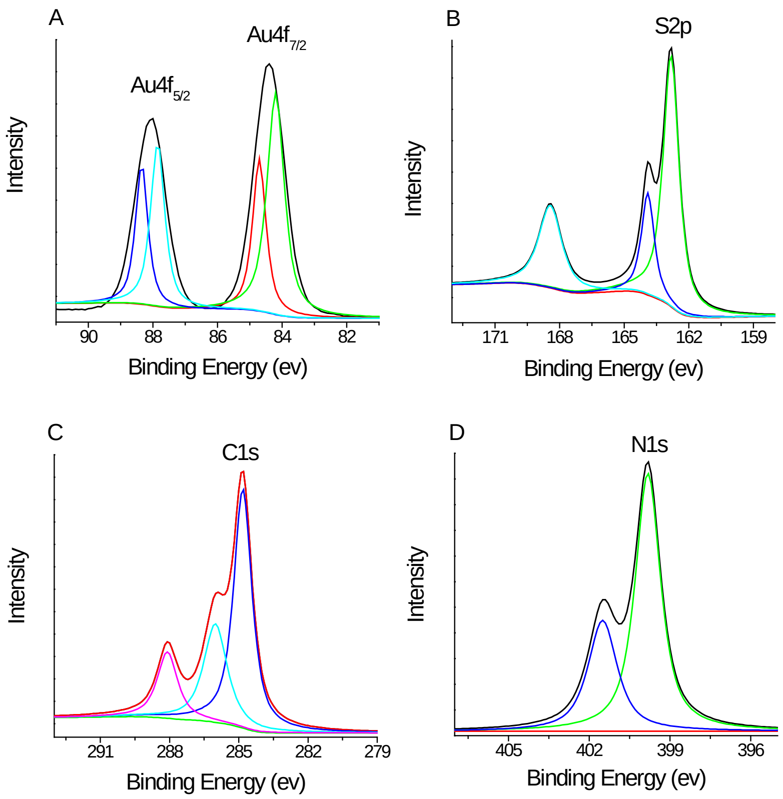

Figure S4. XPS spectra of Au-NC@GSH. (A) The Au $4f_{7/2}$ spectrum could be divided into two distinct components (red and green curves) centered at binding energies of 84.2 eV and

84.7eV, assigned to Au (0) and Au (I), respectively; (B) the bond energy peak of 162.8 eV, 163.9 eV, 170.2eV corresponding to Au-S, S and S-oxide; (C) the bond energy peak 284.8 eV, 286.0 eV and 288.1eV corresponded to ($-CH_2-CH_2-$), ($-CONH_2$) and (-COOH), respectively; (D) the bond energy peaks of 399.8eV and 401.5eV indicate the existence of (-NH) and ($-NH_3^+$). Overall, XPS results indicate Au atoms and Au ions are included in Au-NC@GSH.

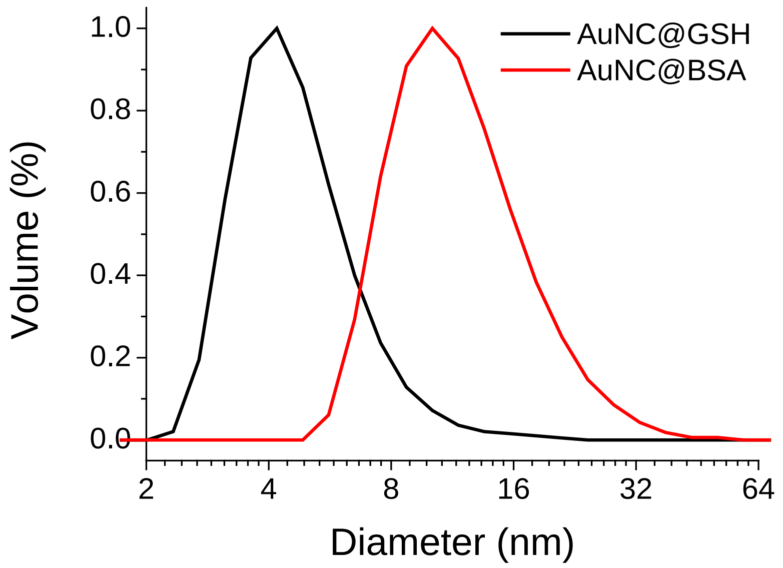

Figure S5. Size distributions of AuNC@GHS and AuNC@BSA dispersed in NF-DMEM culture media. Data were obtained by the dynamic light scattering (DLS) measurements.

Estimation of D_p values through P_{app} values

The P_{app}values are proportional to the $D_p$values as described by the following equation:

$$P_{app} = D_p \cdot \frac{K_{m/f}}{h} = a \cdot D_p$$

Where h is thickness of the membrane and $K_{m/f}$ is a constant for the membrane. Considering that under the condition of complete opening of tight junction (i.e. after treatment with 0.5 mmol/L EDTA), $D_p$would tend to be close to D_{AB}. Therefore, the scale factor a for a certain fluorescent probe can be estimated by the following equation:

$$a = \frac{P_{app,\,max}\,(\text{EDTA treatment})}{D_{AB}}$$

Then the $D_p$values are estimated by the following equation:

$$D_P = \frac{P_{app}}{a}$$

Size exclusion cChromatography of Eu-DTPA and AuNCs

Eu-DTPA (2 µM) and AuNCs (Au-NC@BSA, 1 mg/mL; Au-NC@GSH 0.8 mg/mL) were dissolved in NF-DMEM medium and applied to a Sephadex G25 column (0.5 mL, GE Health Care). The column was eluted with FF-DMEM medium and monitored per 100 µL volume using a *Flexstation 3* microplate reader with a $\lambda_{ex/em}$ of 340/616 nm and a measurement window from 600 to 1000 ms for Eu-DTPA, a $\lambda_{ex/em}$ of 488/630 nm for Au-NC@BSA, and a $\lambda_{ex/em}$ of 405/608 nm for Au-NC@GSH.

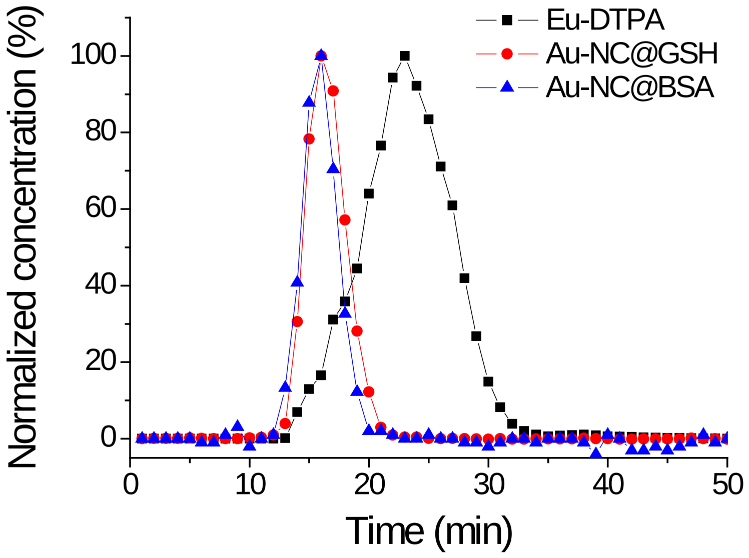

Figure S6. Chromatograms of Eu-DTPA , AuNC@GSH and AuNC@BSA.
